# Artificial intelligence-guided distal radius fracture detection on plain radiographs in comparison with human raters

**DOI:** 10.1186/s13018-025-05888-9

**Published:** 2025-05-16

**Authors:** Nikolai Ramadanov, Patric John, Robert Hable, Andreas Georg Schreyer, Simon Shabo, Robert Prill, Mikhail Salzmann

**Affiliations:** 1 Center of Orthopaedics and Traumatology, Brandenburg Medical School, University Hospital Brandenburg/Havel, 14770 Brandenburg/Havel, Germany; 2 Faculty of Health Science Brandenburg, Brandenburg Medical School Theodor Fontane, Brandenburg/Havel, Germany; 3 Faculty of Applied Computer Science, Deggendorf Institute of Technology, Deggendorf, Germany; 4 Department of Diagnostic and Interventional Radiology, University Hospital Brandenburg, Brandenburg Medical School Theodor Fontane, 14770 Brandenburg, Germany

## Abstract

**Background:**

The aim of this study was to compare the performance of artificial intelligence (AI) in detecting distal radius fractures (DRFs) on plain radiographs with the performance of human raters.

**Methods:**

We retrospectively analysed all wrist radiographs taken in our hospital since the introduction of AI-guided fracture detection from 11 September 2023 to 10 September 2024. The ground truth was defined by the radiological report of a board-certified radiologist based solely on conventional radiographs. The following parameters were calculated: True Positives (TP), True Negatives (TN), False Positives (FP), and False Negatives (FN), accuracy (%), Cohen’s Kappa coefficient, F1 score, sensitivity (%), specificity (%), Youden Index (J Statistic).

**Results:**

In total 1145 plain radiographs of the wrist were taken between 11 September 2023 and 10 September 2024. The mean age of the included patients was 46.6 years (± 27.3), ranging from 2 to 99 years and 59.0% were female. According to the ground truth, of the 556 anteroposterior (AP) radiographs, 225 cases (40.5%) had a DRF, and of the 589 lateral view radiographs, 240 cases (40.7%) had a DRF. The AI system showed the following results on AP radiographs: accuracy (%): 95.90; Cohen’s Kappa: 0.913; F1 score: 0.947; sensitivity (%): 92.02; specificity (%): 98.45; Youden Index: 90.47. The orthopedic surgeon achieved a sensitivity of 91.5%, specificity of 97.8%, an overall accuracy of 95.1%, F1 score of 0.943, and Cohen’s kappa of 0.901. These results were comparable to those of the AI model.

**Conclusion:**

AI-guided detection of DRF demonstrated diagnostic performance nearly identical to that of an experienced orthopedic surgeon across all key metrics. The marginal differences observed in sensitivity and specificity suggest that AI can reliably support clinical fracture assessment based solely on conventional radiographs.

## Introduction

Artificial intelligence (AI) is increasingly used for fracture detection in radiographs, enhancing diagnostic accuracy and efficiency. Studies show AI can perform at a radiologist’s level, aiding fracture detection across various anatomical regions [1, 2]. Machine learning models analyze large datasets, identifying subtle fractures that might not be detetected in human evaluation. While AI reduces workload and speeds up diagnosis, challenges remain, such as reliance on high-quality training data and the risk of misinterpretation. Despite these limitations, AI is becoming a valuable tool in radiology, supporting clinicians in making faster, more reliable decisions—especially in emergency settings.

Distal radius fractures (DRFs) are among the most common fractures in emergency and orthopedic care. Accurate and timely diagnosis is crucial, as missed or misdiagnosed fractures can lead to complications like malunion, chronic pain, and reduced wrist function. AI, particularly deep learning and convolutional neural networks (CNNs), has shown promise in detecting DRFs on radiographs, with numerous studies evaluating different models, their effectiveness, and limitations. Convolutional neural networks (CNNs) are a type of deep learning model designed to analyze images by recognizing visual patterns—such as lines, edges, or shapes—that help identify abnormalities like fractures. These models are trained on large datasets of labeled images and improve through repeated exposure to variations in anatomy and pathology.

Oude Nijhuis et al. [3] developed an open-source CNN for DRF detection, achieving high accuracy (87% internal, 82% external) with an area under the curve (AUC) of 0.93 and 0.88. However, the model’s segmentation performance remained moderate, limiting precise localization of the fracture. Anttila et al. [4] trained a segmentation-based deep learning model on 3,785 radiographs, reaching an AUC of 0.97 and 0.95. While effective, the lack of external validation limits generalizability. Kim et al. [5] compared DenseNet-161 and ResNet-152 (AUC: 0.96 and 0.95) and used activation mapping for interpretability, but external validation was lacking. Oka et al. [6] employed bi-plane radiographs, achieving an AUC of 0.99 on a small dataset—an approach with potential but requiring larger-scale validation. Gan et al. [7] found AI outperformed radiologists and matched orthopedists (AUC: 0.96) but only analyzed AP radiographs, reducing real-world applicability. Suzuki et al. [8] demonstrated near-perfect AI accuracy (99.3%), surpassing specialists, raising concerns about overfitting or dataset limitations. Lee et al. [9] showed AI-guided diagnosis improved novice radiologists’ accuracy, particularly for scaphoid fractures, reinforcing AI’s role as a diagnostic aid. Previous studies often lacked external validation, used small or homogeneous datasets, or focused only on AP radiographs. Unlike many of the previous studies, our investigation is based on a large and diverse real-world dataset, including both AP and lateral view radiographs. Moreover, it directly compares AI performance to that of a human expert in musculoskeletal trauma care, using consistent evaluation metrics and blinded assessment—something few prior studies have done.Ongoing research on AI-guided DRF detection is crucial due to the diversity of AI architectures and training methods. Different models (e.g., CNNs, EfficientNet, DenseNet) perform variably across datasets, complicating direct comparisons. Since no universal AI model exists, further studies are needed to determine which algorithms generalize best across diverse clinical settings. Additionally, external validation remains limited, restricting real-world applicability. Future research should also explore AI integration into clinical workflows, optimizing human-AI collaboration to improve diagnostic accuracy while addressing potential biases and overfitting risks.

This study aimed to compare the performance of AI in detecting DRFs on wrist radiographs with that of human raters.

## Methods

### Study sample

The study was approved by the instutional ethics committee (231072024-BO-E-RETRO), which also waived the requirement for patient informed consent. This retrospective single-center analysis was performed on all wrist radiographs taken between 11 September 2023 and 10 September 2024, following the implementation of AI-guided fracture detection. The inclusion criteria were: (i) patients from all age groups, (ii) radiographs taken in one or two planes of the human wrist, (iii) images with adequate quality and field of view. All straight anteroposterior (AP) and lateral view radiographs were identified from the hospital’s radiographic demonstration program using predefined search parameters based on the body region and examination date.

### Orthopaedic rater evaluation and blinding

All radiographs were reviewed by an experienced orthopedic surgeon (PJ) with over 10 years of clinical and surgical experience in musculoskeletal trauma. The rater was blinded to the study design, patient demographics, and results from both the AI system and the radiologist. Fracture detection findings were recorded in a spreadsheet, alongside the outputs of the AI model and the radiological reports. The diagnostic performance of the orthopedic surgeon was evaluated using the same statistical parameters as for the AI: sensitivity, specificity, accuracy, F1 score, and Cohen’s kappa. This enabled a direct, quantitative comparison between human and AI performance.

### BoneView™ version 2.5.1

Since 11 September 2023, the BoneView™ AI system (version 2.5.1, Gleamer, Paris, France) has been implemented in the clinical practice of our hospital. The AI system automatically assesses each radiograph and generates a result, which is then attached to a copy of the original radiograph for easy access by any involved physician. All radiographs were first interpreted by board-certified radiologists with 1–6 years of post-certification experience in general radiology. These reports were subsequently reviewed and validated by senior radiologists with 10–20 years of experience. While none of the radiologists had formal fellowship training in musculoskeletal imaging, all were routinely involved in trauma radiograph interpretation as part of clinical emergency care.

BoneView™ is a commercially available, Conformité Européenne (CE) marked AI tool designed to assist in detecting fractures, dislocations, effusions, and focal bone lesions in Digital Imaging and Communications in Medicine (DICOM) images. It covers analysis of both upper and lower limbs, the pelvis, thoracolumbar spine, and chest for patients aged 2 years and older. The AI system is based on a CNN built upon Detectron2, an open-source object detection platform developed by Facebook AI Research and implemented with PyTorch (https://pytorch.org/). The training dataset used to develop the algorithm consisted of 500,000 patient radiographs from 22 radiology departments, collected between January 2011 and May 2023.

**Fig. 1:**
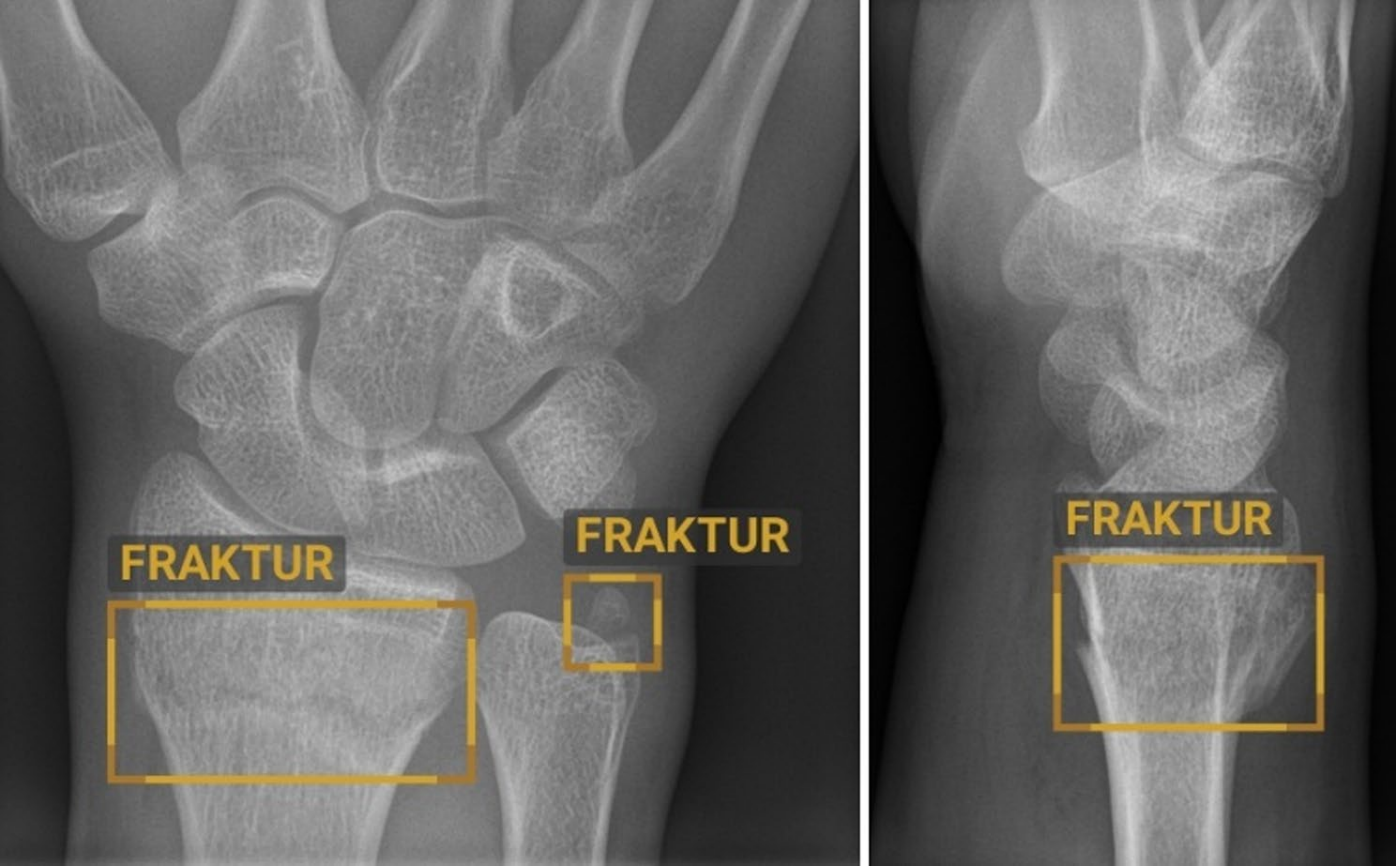
Example of AI-guided fracture detection on wrist radiographs. *AI: artificial intelligence*

The algorithm assigns confidence scores to the radiographs, classifying them as ‘doubtful’ (confidence score between 50% and 90%) or ‘positive’ (confidence score above 90%). Scores below 50% are classified as negative results. These thresholds were derived from the receiver operating characteristic (ROC) curve, optimizing the balance between sensitivity and specificity. The software highlights the region of interest on the radiograph using a rectangular box, with a continuous line for positive results and a dotted line indicating doubt. Figure 1 presents an example of AI-guided fracture detection on wrist radiographs, performed by BoneView™ Version 2.5.1 (Fig. 1).

### Statistical analysis

A professional statistician (RH) performed the statistical analysis using R version 4.2.1. The reference standard (“ground truth”) was defined as the official radiological report issued by a board-certified radiologist, based exclusively on AP and lateral radiographs. The performance of the AI system was evaluated by calculating the following metrics:

- **True Positives (TP)**, **True Negatives (TN)**, **False Positives (FP)**, and **False Negatives (FN)**, derived from a confusion matrix comparing AI predictions to the ground truth.
- **Accuracy** was calculated as the proportion of correctly classified cases (TP + TN) over the total number of cases:
- Accuracy = (TP + TN) / (TP + TN + FP + FN).
- **Cohen’s Kappa coefficient** was used to measure the level of inter-rater agreement between the AI system and human raters, beyond chance:
- κ = (Po - Pe) / (1 - Pe), where Po is the observed agreement and Pe is the expected agreement by chance.
- **F1 Score** was calculated as the harmonic mean of precision and sensitivity:
- F1 = 2 × ((Precision × Sensitivity) / (Precision + Sensitivity)), where Precision = TP / (TP + FP).
- **Sensitivity (Recall)**, or true positive rate, was calculated as the proportion of actual positives correctly identified by the AI:
- Sensitivity = TP / (TP + FN).
- **Specificity**, or true negative rate, was calculated as the proportion of actual negatives correctly identified:
- Specificity = TN / (TN + FP).
- **Youden Index (J Statistic)** was used to summarize the diagnostic effectiveness of the model:
- J = Sensitivity + Specificity − 1.

A p-value calculation was performed for accuracy, sensitivity, and specificity using McNemar’s test, as these standard performance metrics are based on paired binary outcomes and allow for direct statistical comparison. Composite metrics such as Cohen’s Kappa, F1-score, and the Youden Index were reported descriptively, as no standard inferential tests are available for these measures. Importantly, these values are derived from the same underlying contingency table (true positives, false positives, true negatives, false negatives) used in the above p-value calculations, rendering additional significance testing redundant and methodologically inappropriate.

## Results

### Descriptive results

**Fig. 2:**
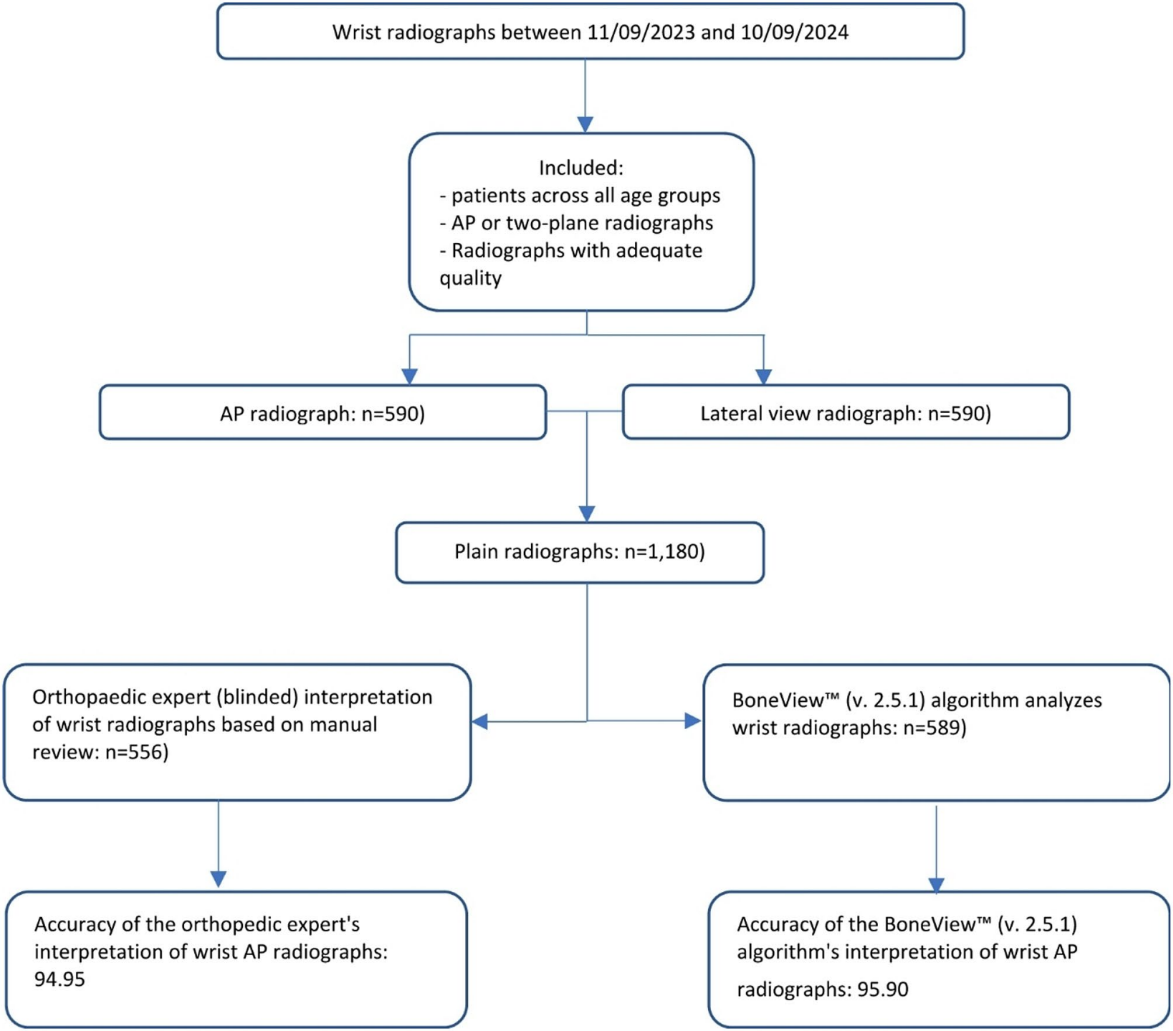
Flowchart diagram. *AP: anteroposterior*

A total of 1,145 plain radiographsof the wrist were, taken between 11 September 2023 and 10 September 2024, were included (Fig. 2). They consist of of 556 AP radiographs and 589 lateral view radiographs. The mean age of the patients included in the study was 46.6 years (± 27.3), with ages ranging from 2 to 99 years. Of the patients, 41.0% were male and 59.0% were female. According to the ground truth, of the 556 AP radiographs, 225 cases (40.5%) showed a DFR, while 240 cases (40.7%) of the 589 lateral view radiographs revealed a DFR. A descriptive analysis of the included radiographs is shown in (Table 1).

**Table 1:** Descriptive analysis of the included radiographs. *SD: standard deviation; AP: anteroposterior*

Parameter	Value
Patients (N)	590
Sex (N,%)	male: 242 (41.0%) female: 348 (59.0%)
Age (years ± SD; min.– max.)	46.6 ± 27.3; 2.0-98.8
Total number of radiographs (N)	1145
AP radiograph (N)	556
Fracture (N,%)	Ground truth No: 331 (59.5%)
	Ground truth Yes: 225 (40.5%)
Lateral view radiograph	589
Fracture (N,%)	Ground truth No: 349 (59.3%)
	Ground truth Yes: 240 (40.7%)

### Statistical analysis

#### Accuracy

**Fig. 3:**
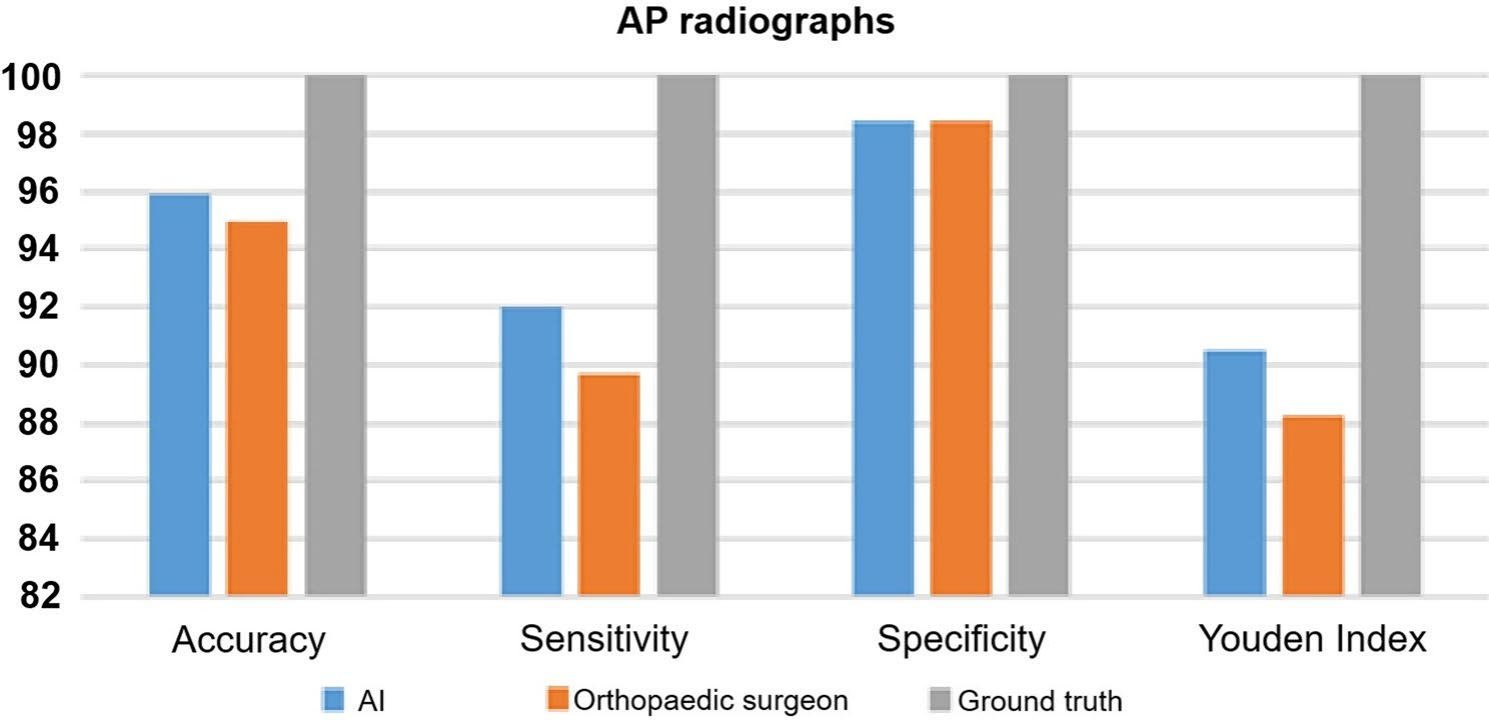
AI performance on AP radiographs for accuracy, sensitivity, specificity and Youden Index compared to human raters. *AI: artificial intelligence; AP: anteroposterior*

**Fig. 4:**
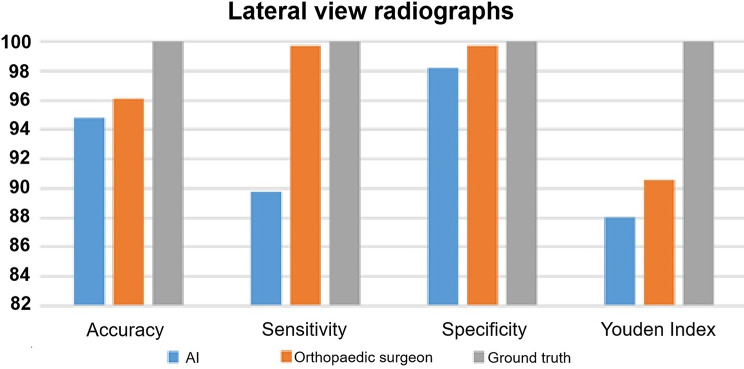
AI performance on lateral view radiographs for accuracy, sensitivity, specificity and Youden Index compared to human raters. *AI: artificial intelligence*

The accuracy (%) of AI was 95.90 on AP radiographs and 94.81 on lateral view radiographs (Fig. 3; Table 2). The accuracy (%) of the orthopedic surgeon was 94.95 on AP radiographs and 96.10 on lateral view radiographs (Fig. 4; Table 2).

**Table 2:** Statistical analysis of AI performance compared to human raters

	AI on ap radiograph	Orthopaedic surgeon on ap radiograph	*p*-value	AI on lateral view radiograph	Orthopaedic surgeon on lateral view radiograph	*p*-value
Accuracy	95.90	94.95	0.239	94.81	96.10	0.646
Cohen’s Kappa	0.913	0.894	N/A	0.891	0.918	N/A
F1 score	0.947	0.935	N/A	0.934	0.950	N/A
Sensitivity	92.02	89.73	0.182	89.79	90.83	0.773
Specificity	98.45	98.48	1.000	98.25	99.71	0.131
Youden Index	90.47	88.22	N/A	88.04	90.55	N/A
True Positives	196	201	N/A	211	218	N/A
True Negatives	318	325	N/A	337	348	N/A
False Positives	5	5	N/A	6	1	N/A
False Negatives	17	23	N/A	24	22	N/A

AI: artificial intelligence; AP: anteroposterior; N/A: not applicable

### Cohen’s kappa

**Fig. 5:**
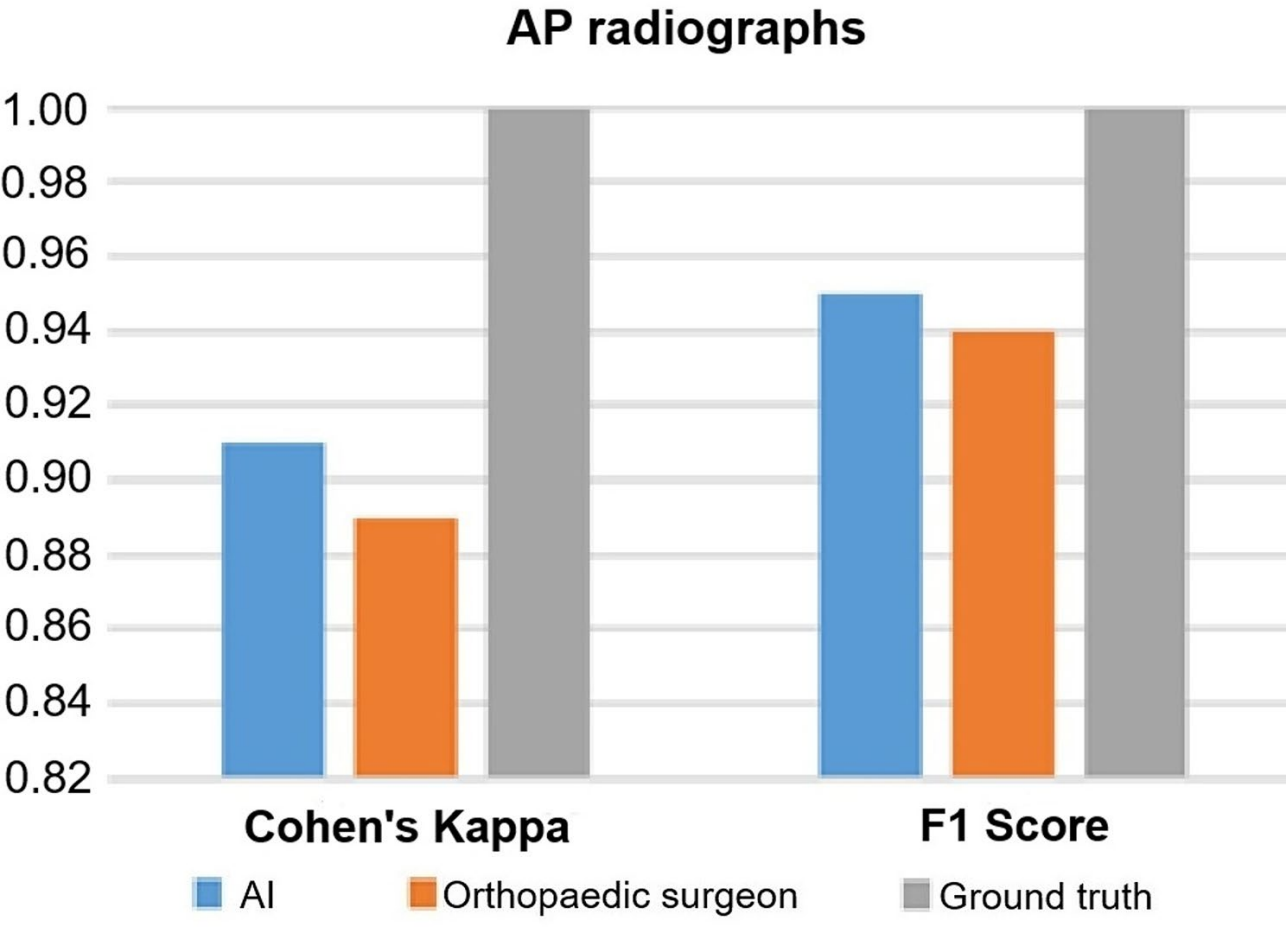
AI performance on AP radiographs for Cohen’s Kappa and F1 score compared to human raters. *AI: artificial intelligence; AP: anteroposterior*

**Fig. 6:**
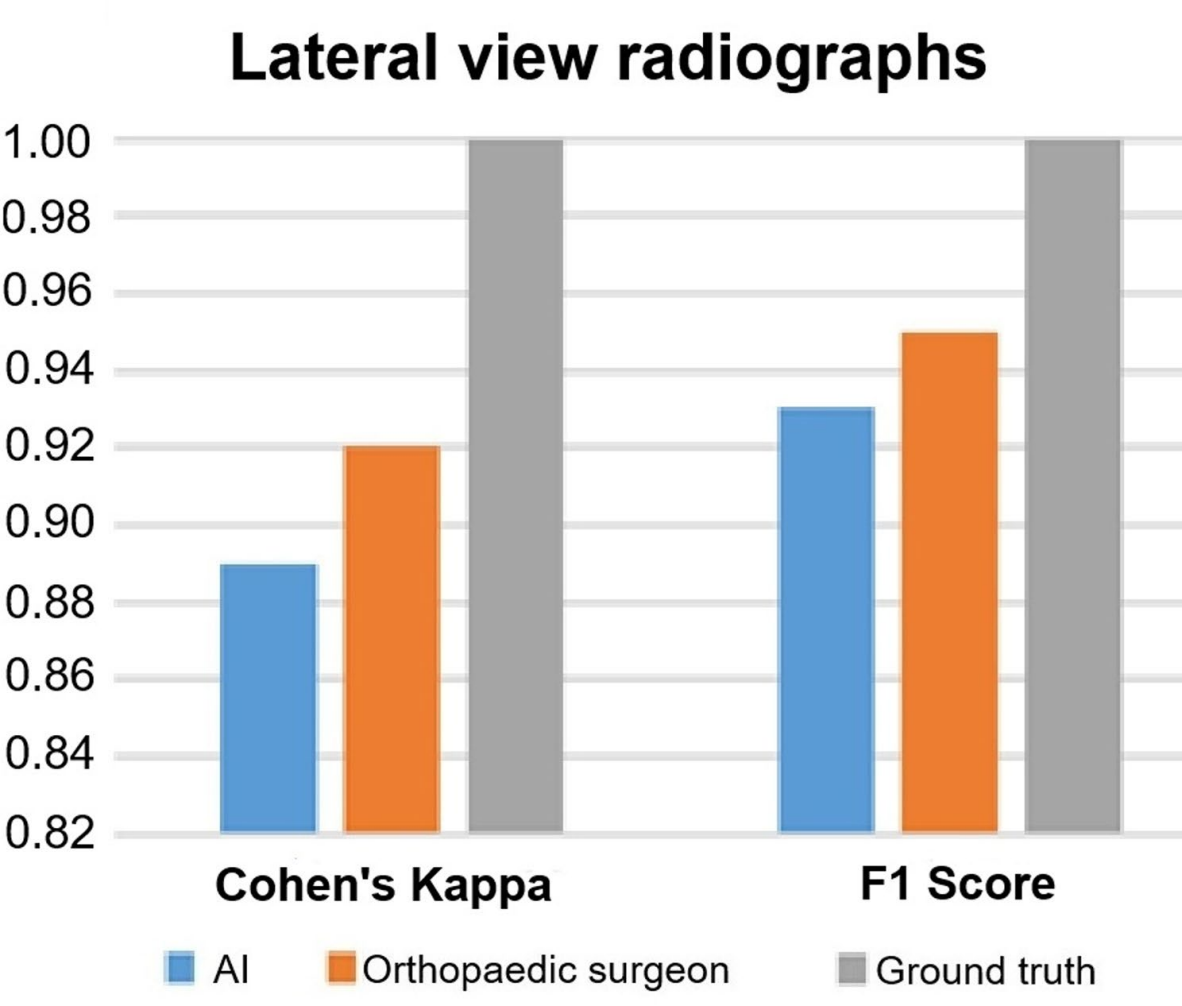
AI performance on lateral view radiographs for Cohen’s Kappa and F1 score compared to human raters. *AI: artificial intelligence*

Cohen’s Kappa of AI was 0.91 on AP radiographs and 0.89 on lateral view radiographs (Fig. 5; Table 2). Cohen’s Kappa of the orthopedic surgeon was 0.89 on AP radiographs and 0.92 on lateral view radiographs (Fig. 6; Table 2).

### F1 score

F1 score of AI was 0.95 on AP radiographs and 0.93 on lateral view radiographs (Fig. 5; Table 2). F1 score of the orthopedic surgeon was 0.94 on AP radiographs and 0.95 on lateral view radiographs (Fig. 6; Table 2).

### Sensitivity

The sensitivity (%) of AI was 92.02 on AP radiographs and 89.79 on lateral view radiographs (Fig. 3; Table 2). The sensitivity (%) of the orthopedic surgeon was 89.73 on AP radiographs and 99.71 on lateral view radiographs (Fig. 4; Table 2).

### Specificity

The specificity (%) of AI was 98.45 on AP radiographs and 98.25 on lateral view radiographs (Fig. 3; Table 2). The specificity (%) of the orthopedic surgeon was 98.48 on AP radiographs and 99.71 on lateral view radiographs (Fig. 4; Table 2).

### Youden index

The Youden Index of AI was 90.47 on AP radiographs and 88.04 on lateral view radiographs (Fig. 3; Table 2). The Youden Index of the orthopedic surgeon was 88.22 on AP radiographs and 90.55 on lateral view radiographs (Fig. 4; Table 2).

To provide a direct comparison of diagnostic performance, Table 2 summarizes the key metrics for both the AI system and the orthopedic surgeon on AP radiographs.

## Discussion

### Main findings

The most important finding is that AI-guided detection of DRFs on radiographs is highly accurate, achieving performance comparable to that of experienced orthopedic surgeons. The AI system exhibited high sensitivity (92.02% for AP radiographs and 89.79% for lateral view radiographs) and specificity (98.45% and 98.25%, respectively), with an overall accuracy exceeding 94% in both planes. These findings highlight the potential of AI as a reliable diagnostic support tool in clinical settings.

The present study demonstrates high accuracy and reliability of AI-guided DRF detection. A comparison with previous studies [3–9] highlights both similarities and differences in methodologies, validation approaches, and clinical applicability. Oude Nijhuis et al. [3] developed an open-source CNN for DRF detection, achieving an internal accuracy of 87% and external accuracy of 82%. Compared to our study, their external validation results indicate lower performance, which may be attributed to dataset heterogeneity. Additionally, their fracture localization accuracy remained moderate (AP50: 29–25), whereas the AI system demonstrates superior sensitivity and specificity. Anttila et al. [4] utilized a segmentation-based deep learning model trained on 3,785 radiographs, achieving an AUC of 0.97 and 0.95. While this study demonstrated excellent performance, it lacked external validation. Our study, by comparison, offers clinically validated results, reinforcing AI’s reliability in a real-world setting. Kim et al. [5] applied DenseNet-161 and ResNet-152 models, achieving AUCs of 0.962 and 0.947, respectively. Our study’s results align closely, with comparable sensitivity and specificity, suggesting that different CNN architectures may perform similarly when trained with adequate datasets. Oka et al. [6] introduced a bi-plane radiograph approach, achieving an exceptionally high AUC of 0.991 despite using a relatively small dataset. This suggests that bi-plane imaging may enhance AI performance, a potential avenue for future refinement of the AI model. Gan et al. [7] compared AI performance against radiologists and orthopedists, finding that AI outperformed radiologists but matched orthopedists (AUC: 0.96). Our study corroborates this finding, as AI achieved performance comparable to an experienced orthopedic surgeon. Suzuki et al. [8] reported near-perfect AI accuracy (99.3%), surpassing hand orthopedic surgeons. Such high accuracy raises concerns of overfitting, whereas our study’s balanced sensitivity and specificity suggest a more generalized model. Lee et al. [9] assessed AI in detecting multiple wrist fractures, emphasizing improved diagnostic accuracy for novice radiologists. Similarly, our study highlights AI’s role in augmenting human expertise rather than replacing it.

The broader landscape of AI applications in fracture detection extends beyond DRFs, as highlighted in several comprehensive reviews. Ashworth et al. [10] emphasize the rapid advancements in AI-based pediatric fracture detection, yet they note that while AI models show high diagnostic accuracy, significant gaps remain in clinical validation, cost-effectiveness, and bias assessment. This aligns with the need for AI tools to undergo rigorous real-world evaluation before widespread clinical integration. Ghasemi et al. [11] provide a meta-analysis on AI-driven osteoporosis detection using panoramic radiographs. Their findings suggest that while AI demonstrates high sensitivity (87.92%) and specificity (81.93%), heterogeneity in study designs and potential small-study effects may influence reported accuracy. This underscores the necessity for larger, standardized datasets to improve AI reliability across different clinical settings. Binh et al. [12] examine AI’s role in pediatric elbow fracture detection, where deep learning models achieved an AUC of 0.95. Their analysis highlights the importance of selecting appropriate backbone architectures, such as ResNet, to optimize AI performance. This review also stresses that manual preprocessing by radiology experts remains a critical factor in enhancing AI-based fracture detection. Collins et al. [13] focus on AI’s role in rib fracture detection via radiograph and computed tomography (CT) imaging, revealing that AI achieved higher sensitivity (0.84) compared to radiologists (0.75). This suggests that AI can not only assist but potentially outperform human experts in specific diagnostic tasks, particularly when rapid interpretation is needed in emergency settings. Lo Mastro et al. [14] discuss AI’s general impact on fracture detection, emphasizing its ability to standardize interpretations across radiologists with varying levels of experience. Structured AI-generated reports, they argue, can reduce variability in diagnoses and enhance workflow efficiency, a key advantage in high-throughput radiology departments. Kutbi [15] expands the discussion beyond fracture detection, exploring AI applications in 3D CT and magnetic resonance imaging (MRI). Additionally, the review highlights the potential of generative AI and large language models to refine diagnostic capabilities through synthetic data generation and automated report creation. However, ethical considerations and model robustness remain crucial challenges that must be addressed before AI can achieve full clinical acceptance.

### Clinical implications

The implementation of AI in fracture detection could significantly enhance diagnostic efficiency in emergency and orthopedic settings. By reducing radiologists’ workload and providing rapid, reliable assessments, AI can assist in early and accurate diagnosis, potentially leading to improved patient outcomes. Moreover, AI can serve as a valuable tool in resource-limited environments where access to experienced radiologists may be restricted. The high specificity of our AI model also suggests a low risk of unnecessary follow-up imaging or interventions, which could optimize healthcare resource utilization.

Beyond its role in diagnostic support, AI has the potential to improve triage workflows by prioritizing suspected fractures for radiologist review, thereby reducing delays in patient management. Additionally, AI could be integrated into telemedicine frameworks, allowing remote evaluation of radiographs in underserved areas where access to orthopedic specialists is limited. Another promising application is in medical education, where AI-guided tools can help train radiology and orthopedic residents by providing immediate feedback on fracture detection and classification. However, successful implementation requires careful consideration of ethical and medico-legal aspects, including liability for AI-driven misdiagnoses and the need for continuous monitoring of AI performance to prevent biases from influencing clinical decisions. Future research should focus on optimizing AI integration into clinical workflows to maximize its benefits while mitigating potential risks.

### Limitations and strengths

One limitation of our study is its retrospective nature, which may introduce selection bias. Additionally, our dataset consists of radiographs from a single institution, limiting external generalizability. Another limitation is that the ground truth was based solely on radiologist interpretation of radiographs, which, although reflective of clinical practice, may be prone to occasional diagnostic error. The AI system’s performance was evaluated against ground truth based on radiological findings, which, while reliable, may not fully account for all clinical variables influencing fracture diagnosis. Furthermore, although AI exhibited high accuracy, there were still false positives and false negatives, indicating that AI should be used as an adjunct rather than a replacement for human expertise.

A strength of our study is its relatively large sample size and the inclusion of both AP and lateral view radiographs, ensuring a robust evaluation of AI performance. The comparison with an experienced orthopedic surgeon provides a clinically relevant benchmark, and the use of multiple statistical metrics enhances the reliability of our findings.

## Conclusion

AI-guided detection of distal radius fractures is highly accurate and comparable to human expert evaluation. AI has the potential to improve diagnostic efficiency and support clinicians in DRF assessment. However, further research is needed to validate AI performance across diverse clinical settings, different fractures, and to explore its integration into routine workflows. At the moment, AI should be viewed as a complementary tool that enhances, rather than replaces, human expertise in DRF diagnosis.

## Data Availability

Available upon reasonable request.

## Abbreviations

AI: Artificial intelligence
AUC: Area under the curve
AP: Anteroposterior
CE: Conformité Européenne
CT: Computed tomography
CNN: Convolutional Neural Network
DICOM: Digital Imaging and Communications in Medicine
DRF: Distal radius fractures
FN: False negative
FP: False positive
MRI: Magnetic resonance imaging
ROC: Receiver operating characteristic
TP: True positive
TN: True negative
F1: F1 Score
Po: Observed agreement
Pe: Expected agreement by chance
